# Perspective of parents on healthcare for children during COVID-19 in India: A qualitative inquiry

**DOI:** 10.6026/973206300220362

**Published:** 2026-01-31

**Authors:** Hepsi Bai Joseph, Sandhiya Kuppusamy, Santosh Kumar Mahalik, Asha P Shetty, Kanishka Das

**Affiliations:** 1 Department of Pediatric Nursing, College of Nursing, AIIMS, Bhubaneswar, Odisha, India; 2 Department of Pediatric Surgery, All India Institute of Medical Sciences, Bhubaneswar, Odisha, India

**Keywords:** COVID-19 pandemic, health-seeking behaviour, parents' perspectives, challenges, India

## Abstract

The COVID-19 pandemic had pushed everyone into isolation at home and created challenges for mankind to meet their healthcare needs. Therefore, it was of interest to explore the perspectives of parents in caring for children with healthcare needs during the COVID-19 pandemic in India. An exploratory qualitative inquiry was conducted among 16 parents through in-depth interviews at a tertiary care hospital using a purposive sampling technique. The current study found two themes: disablers towards the health facility approach and circumstances-induced challenges. These alarming facets of the health delivery system warrants introspection on the possibilities of rendering better services to vulnerable groups by creating awareness and alternative facilities. Moreover, the health ministry and related stakeholders need to plan improved facilities and alternative channels to manage health needs and prevent such experiences during future pandemics.

## Background:

The World Health Organisation declared COVID-19 as a global pandemic on March 11th, 2020. To contain the virus and prevent its spread, the World Health Organisation recommended preventive measures, including social distancing, avoidance of crowding, and staying at home [1]. These restrictions were implemented in India by imposing a nationwide lockdown, which shut educational institutions at the school and college levels, as well as micro and macro industries and general transportation services [2]. Additionally, most private and public health facilities had shut down their regular healthcare services, including Outpatient Department services, elective surgeries, and community outreach activities, and catered primarily to emergency surgeries and COVID-19 services. Most inpatient and intensive care units were converted into COVID care units to meet the exclusive health needs of patients [3]. With regard to health-seeking behaviour, patients with chronic disease requiring follow-up care, both adult and pediatric, suffered due to the pandemic lockdown. Children are a vulnerable population; their health needs were not met, especially those with chronic diseases, oncological conditions, surgical needs, as well as follow-up care. Studies found missed follow-up, missed medication, and death as the consequences of the pandemic [4]. von Rhein *et al.* identified a reduction in the utilization of pediatric emergency care. All these changes were due to a lockdown imposed by the government to prevent overcrowding and contain the spread of the virus [5]. The challenges experienced by parents have not been extensively explored; few studies have dwelled on them quantitatively or retrospectively, but not qualitatively. In the vulnerable pediatric population, there is a lack of evidence on how parents accessed their healthcare services during the COVID-19 pandemic. Therefore, it was of interest to explore the perspectives of parents in meeting their children's healthcare services during the COVID-19 pandemic using a qualitative approach.

## Methodology:

## Setting and participants:

The present study was conducted at a tertiary public hospital in Eastern India with specialty & super-specialty departments that care for patients. During the COVID-19 pandemic, outpatient department services were shut down to contain the spread of the virus and prevent overcrowding. Only emergency patient services, including surgeries and COVID care services, were performed. The inpatient department was converted into COVID care wards and COVID ICUs, retaining 30% of the beds for other illnesses to support pediatric and adult services. The pediatric department halted its regular services and focused on emergency interventions for the sick. The present study examined the parents' perspectives on approaching health services during the COVID-19 pandemic with a qualitative approach. In- depth interviews were conducted on 16 parents using a purposive sampling technique. A semi-structured interview schedule was used to collect the data. Ethical permission was obtained from the Institutional Ethics Committee of All India Institute of Medical Sciences, Bhubaneswar, India. Written consent (face-to-face interview) was obtained from each participant before initiating the interview.

## Data collection methods:

The study employed a qualitative approach and conducted face-to-face, individual interviews using a semi-structured interview schedule. Data was collected through in-depth interviews (IDIs). The investigator conducted face-to-face interviews (16 interviews) with the parents of children who were admitted to the pediatric ward and had undergone emergency intervention for disease conditions during the pandemic lockdown. The interview was conducted in the local language (Odia). After receiving informed written consent, the socio- demographic and clinical characteristics of the participants were collected. The participants were aware that the interview session was audio recorded, and confidentiality was ensured. The interview was conducted using open-ended questions and lasted 20 - 45 minutes. The in-depth interview guide contained the following questions:

## Box 1. Semi-structured interview guide:

[1] Explain how you brought your child during the COVID-19 pandemic?

[2] Detail the experiences and challenges you encountered in accessing this health care facility.

[3] Explain the differences in facilities that you felt before and during the COVID-19 pandemic.

[4] How would you describe the in-hospital experience in caring for your child?

[5] Explain the support services you received in meeting the health care needs of your child.

As the parents discussed how they could or could not meet the health facility's requirements for availing the child care services and the challenges they faced in approaching the healthcare facility, the investigator asked questions to help the participants explain whether the children's healthcare needs were being met or not. Once the data was saturated after the 16th interview, no further information was added from the participants, and the interviews were terminated.

## Data analysis:

The digitally recorded interviews were initially transcribed verbatim and then translated into English. The data were subjected to thematic analysis [6]. The first author performed the coding process and the second author confirmed the codes, themes and sub-themes. The data was systematically analyzed, and the transcripts were read and re-read several times, which provided an overall understanding of the researcher's content. Initial codes were then generated and allocated to relevant sentences and paragraphs, which helped identify the concepts. Later, the concepts were grouped into themes. The themes were reviewed and named, along with their definitions. The themes were primarily descriptive and represented a broad scope, allowing for variation. Instead of imposing a preselected theoretical grid on the data, this method ensured that the coding frame elements reflected the language of parents' perspectives and challenges towards meeting children's healthcare services during the COVID-19 pandemic lockdown. Data management was performed by MAXQDA software.

## Trustworthiness/Rigor:

Credibility was ensured by including study participants with varied clinical characteristics and employing a medical follow-up approach at the healthcare facility. The authors are from diverse educational backgrounds with experience in both academic teaching and research; this has enriched the study's findings and facilitated data triangulation. During analysis, the audiotaping of interviews and auditing of transcripts enhanced the dependability of the data. The researchers' condensation of interviews, thick description of data and exploration of similarities and differences in parents' experiences of their children's healthcare needs were conducted independently. We used the Consolidated Criteria for Reporting Qualitative Studies (COREQ) guidelines for reporting our study [7].

## Results and discussion:

**Figure 1:**
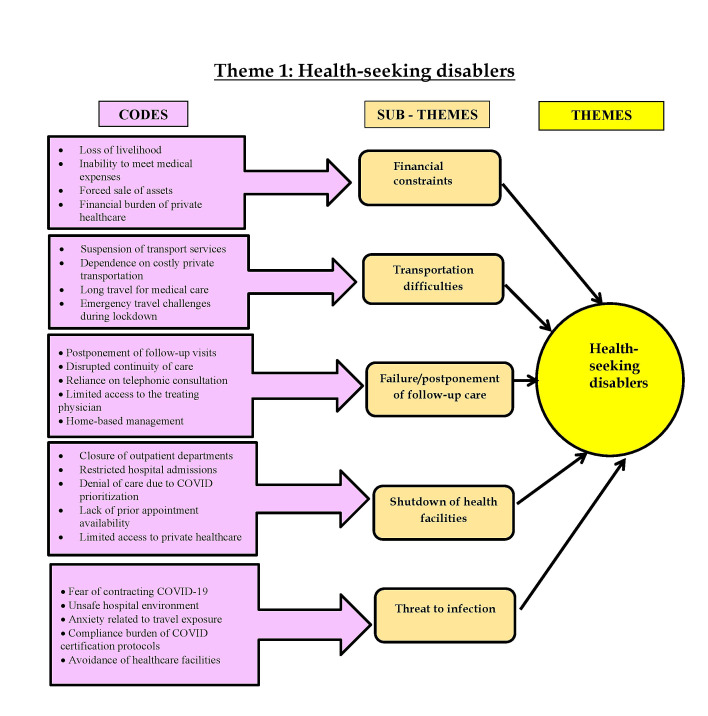
Codes, subthemes of theme 1- Health-seeking disablers

**Figure 2:**
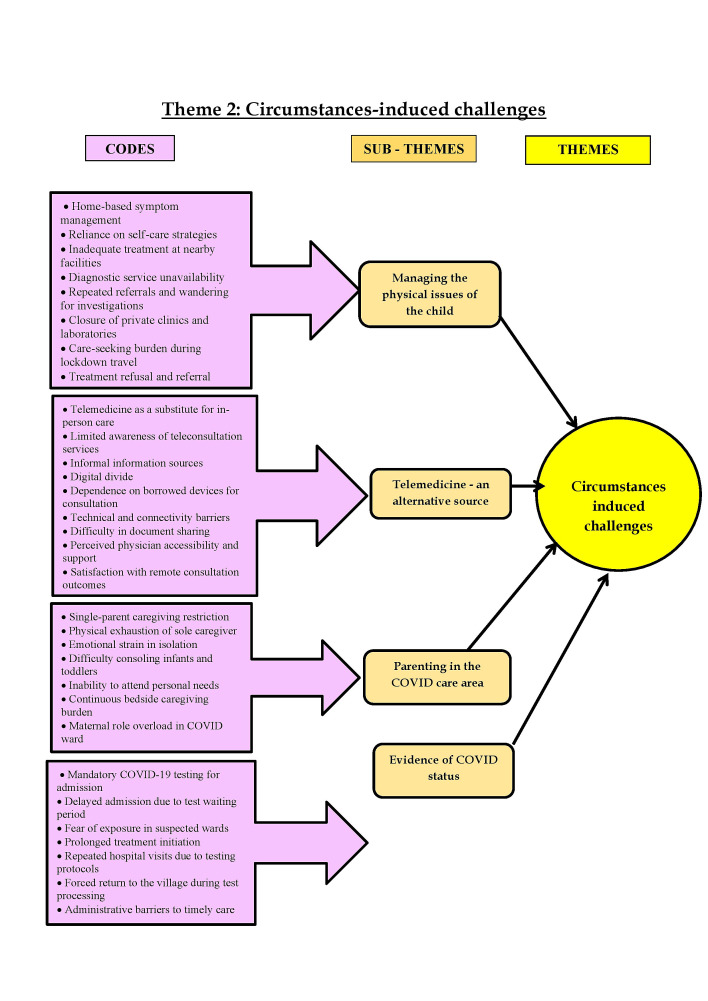
Codes, subthemes of theme 2- circumstances induced challenges

The mean age of the parents was 35±5 years. The majority (80%) of them were mothers with primary school as their educational status and homemakers. The mean age of the children was 8 ± 3 years. The study found two themes, including "Health-seeking disablers and pandemic circumstances induced challenges". The codes, subthemes, and themes are shown in Figure 1 and Figure 2. The description of the results is presented in detail below.

## Health-seeking disablers:

## Financial constraints:

Parents reported that the pandemic shattered their lives by losing or difficulty in attending jobs due to lockdown, resulting in financial scarcity to meet the child's health needs. Parents searched for financial alternatives or opportunities to manage their lives and meet the child's health needs. A parent specified that he had to sell his property to finance his child's medical intervention in a private hospital during the lockdown, as they were unable to reach the public healthcare facility that was too far away.

*"I could not go to work for two months. For my child's treatment, I sold a land of mine to meet all the financial needs" (Parent 1)*

## Transportation difficulties:

Parents reported that due to the nationwide lockdown, transportation services were discontinued and it affected their decision to seek higher-level healthcare facilities. Instead, they accessed nearby healthcare facilities that could not resolve or treat the child's illnesses. The only option was to hire private transportation services that cost twice or thrice. Most of the children who underwent surgery were from distant places; it was challenging to complete follow-up as they had to travel more than 100 kilometres for it. The parents of children who needed emergency admission for critical interventions took great efforts to travel to get inpatient care for their children.

*"As it was a lockdown and no public transport facility was available nearby, I took private transport to reach the hospital" (Parent 2)*

## Shutdown of health facilities:

The OPD services were suspended to contain the spread of the virus, prevent overcrowding, protect healthcare workers from the pandemic and focus their services on COVID-19 patients. Despite all the difficulties, parents approached the healthcare facility but were disappointed to hear about the cessation of outpatient services and had to wait for a physician without a prior appointment. Additionally, most private hospitals and diagnostic centers also suspended their services due to the spread of the pandemic.

*"Initially, my child was not allowed admission in any hospital, they said that that there was no provision to admit due to COVID, we did not know if any doctor would also recommend." (Parent 6)*

## Threat to infection:

Parents of children with follow-up care needs mentioned that they had postponed the child's follow-up care due to the lockdown as the hospital outpatient services had shut down. Besides, parents were unwilling to approach health care facilities for various reasons, including fear of contracting the COVID-19 infection by their children in the hospital or during travel to the health facility in the pandemic environment that might worsen the clinical condition. As per government protocol, the parents were also expected to show their COVID-free status at each state/ district border - this deterred them from seeking healthcare for their child's illness at a distant facility and they preferred managing with nearby health resources.

*"Because of the COVID pandemic, it was not safe to take the child to the hospital and outside environment" (parent-8)*

## Failure/postponement of follow-up care:

Parents of children with post-operative follow-up care acknowledged that they had postponed their children's follow-up visits due to the COVID-19 pandemic. They also expressed that they could approach the physician through telephone. A few parents reported that they were unable to connect with the physician because they lacked access to either the hospital or the physician and therefore managed the child at home.

*"The doctor asked to come for a follow-up after seven days of surgery, but due to COVID, we could not come" (parent 10)*

## Circumstances induced challenges:

## Managing the physical issues of the child:

Parents reported that they self-managed the child's physical illnesses, like pain and diarrhea, with home-based resources. Few parents took their children to a nearby public health facility, where they could not be diagnosed or treated, and had to wander for diagnostic investigations.

As most private clinics and labs were not functional, parents had to scout for alternatives and travelled amidst the pandemic with the sick child - this was shared as a distressing experience. Inpatient treatment refusal and referral services were experienced by parents who came from faraway places for their child's treatment.

*"Due to COVID, scan and testing were closed. So we wandered to other testing facilities with the child in this COVID situation." (Parent-15)*

## Telemedicine - an alternative source:

Telemedicine was considered a vital anchor for parents to manage their children's healthcare needs. A few parents mentioned that they were not aware of Telemedicine and had not been apprised. Additionally, a few individuals received information on telemedicine facilities from their neighbours, social media groups, or physicians. The limiting factor was that many parents did not possess smartphones with internet facilities to utilize such facilities. Despite this, they borrowed gadgets to share the lab investigation reports and other documents related to the child's illness. Technical difficulties, including network connectivity failures and poor access, hindered the physician's ability to consult. On the other hand, parents who accessed through Telemedicine felt satisfied with the response, guidance, and prescription given by the physician and conceded that they were approachable and provided timely support.

*"Many times I tried to connect to the telemedicine facility and in the beginning; it was difficult to connect due to technical and internet issues." (Parent 7)*

*I did not have a smartphone with me. The one who dropped us (cab driver) clicked my child's report and helped to share it with the doctor through WhatsApp." (parent 13)*

## Evidence of COVID status:

Parents expressed that every child who had to undergo medical interventions needed to prove themselves to be COVID-free before admission. Hence, after submitting the COVID test, they waited in the 'suspect ward' until the results were available. Parents were worried that the child would contract COVID-19 from the 'suspect ward', as all patients were retained there till the results were declared. Parents reported that hotels were closed due to lockdown protocol to prevent the spread of COVID-19 and they had to return to their village after consulting the physician and providing the COVID sample. They reported back at the hospital once the child had a proven negative status for admission and further treatment which were thereby prolonged.

*"Until the COVID test report came, I waited outside the hospital and after showing the COVID result as negative, my child got admitted." (Parent 5)*

## Parenting in the COVID care area:

Parents also expressed that it was a great challenge to care for the children in the COVID ward, as only one parent was permitted to stay with the child to avoid COVID-19 spread. Most mothers who accompanied their infants / toddlers with COVID-positive states struggled to care for and console them.

*"I was alone to care for my baby and as it was restricted to one parent per patient in the COVID unit, it was not possible to leave the child alone. I could move out of bed once the child was resting" (Parent-4).*

## Discussion:

In the present study, every parent experienced one or more challenges as they struggled at various points during their journey to the healthcare facility and within the facility itself. Inequalities in the Indian healthcare system, social disparities and a faltering economy had made the pandemic lockdown a veritable threat to the poorer section of society. With difficulty, the parents had approached private transportation services and a few used their vehicles to travel to the hospital to meet their children's healthcare needs. Fazio *et al.* stated that travel restrictions and reduced mobilization elevated the risks of inadequate access and utilization of essential health services, particularly affecting vulnerable groups including children, during the COVID pandemic [8]. This finding is echoed in another study from Kenya on the health-seeking behavior of pregnant mothers during the COVID-19 pandemic; they identified three themes: delays in deciding to seek care, delays in reaching healthcare facilities and delays in receiving quality healthcare services [9]. The delays were due to uncertainty about the COVID-19 pandemic, loss of income for many households, the influence of traditional birth attendants, compulsory lockdowns, and national cessation of movement and compulsory quarantine. The participants' personal experiences therein are consistent with Indian situations. Healthcare institutions had shut down outpatient services, elective surgeries, and other peripheral community services to prevent the spread of the virus and avoid overburdening healthcare team members in the hospital, while caring for COVID-19 patients. Likewise, Vrijlandt *et al.* from the Netherlands reported a reduction in pediatric emergency service utilization, inpatient admissions and outpatient visits [10]. A retrospective cohort study conducted by Mitura *et al.* found an abrupt reduction in pediatric healthcare utilization due to the COVID-19 pandemic lockdown [11]. In India, as the initial phase of COVID-19 prompted stakeholders to implement strict policies to ensure a COVID-free status report for admission/interventions, parents struggled to obtain the same and the consequent postponement of follow-up care for their children. In addition, due to fear of the spread of COVID infection and increasing restrictions, most parents avoided taking risks and managed the children's physical symptoms by consulting nearby health clinics than traveling to the tertiary care health facility. A similar finding was reported in a qualitative study conducted in Nepal, where participants perception of a risk of contracting an infection while accessing healthcare facilities hindered their follow-up care. It had also affected their health-seeking behavior [12]. The public in Nepal was unable to connect to healthcare facilities due to a transportation halt. Only a few could connect with mobile phones, much like the Indian scenario.

In the hospital, the pandemic led to a decline in family-centered care compared to the pre-pandemic situation. Since the risk of COVID-19 transmission from infected children to family members was high, only one parent/caregiver was permitted to stay with the child throughout the COVID treatment, and it was challenging for the sole caregiver. A strict policy on visitor control was implemented in each ward, and parents were required to have a second person accompanying them to stay elsewhere while caring for their child. Krewulak *et al.* stated that to maintain public safety, it was necessary to restrict the physical presence of family members of hospitalized children [13]. These pieces of evidence imply that India stands at a critical juncture, where it must rethink its approach to public health and prepare for future pandemics. A study conducted in Uttar Pradesh, a populous state of India, on the utilization of health and nutrition services found that the service utilization was reduced by 80% and the public faced challenges in limited travel, non-availability of medical services, and fear of getting infected during travel to meet the health care provider [14]. A key strength of this study lies in its qualitative, exploratory approach, which provided rich, in-depth insights into the lived experiences and perspectives of parents navigating healthcare challenges for their children during the COVID-19 pandemic. The use of face-to-face, in-depth interviews allowed for a nuanced understanding of their struggles, emotions, and adaptive behaviors. Employing purposive sampling ensured that participants directly affected by the issue were included, enhancing the relevance and applicability of findings.

## Conclusion:

We report the challenges faced by parents of children with healthcare needs during COVID-19 in India. This includes financial constraint, transport restrictions, limited access to timely care, and heightened psychological stress. While telemedicine emerged as an interim solution, it exposed both gaps and untapped potential in digital health delivery for pediatric care. Data shows the need for policy action, stronger health systems, and parent-centered support to ensure continuity of pediatric care during future public health emergencies.
